# AIDS epidemic at age 25 and control efforts in China

**DOI:** 10.1186/1742-4690-3-87

**Published:** 2006-12-01

**Authors:** Yiming Shao

**Affiliations:** 1 National Center for AIDS/STD Control and Prevention, Chinese Center for Disease Control and Prevention, 27 Nanwei Road, Xuanwu District, Beijing, China

## Abstract

In the first 10 years of AIDS epidemic in China, intravenous drug users (IDUs) and Former Plasma Donors (FPDs) were hardly hit in the late 1980s and mid 1990s respectively. In the last 10 years, while IDU epidemic keeps at a fast pace, sexual transmitted cases of HIV have been steadily increasing. All signs indicate that the HIV epidemic in China is at a turning point, spreading from high risk groups to the general population. Learning from the SARS epidemic, China has recently launched an impressive AIDS campaign by making serious political commitments, and by strengthening the public health system and implementing an aggressive Four Free One Care Policy. There remains huge challenges both at the societal level which form the roots of the AIDS epidemic and at increasing the capabilities of the implementation teams. In addition to other needed efforts, enhancing AIDS research through international collaborations will strengthen China's ability to conduct her huge control program efficiently. Only with a scientific approach and evidence-based strategy, can China seize the opportunity to stop AIDS at an early stage.

## The history and current status of the HIV/AIDS epidemic in China

China reported its first HIV/AIDS cases among hemophiliac patients in Zhejiang province in 1985 [1]. In the same year, a foreigner was diagnosed with AIDS in Beijing [2]. There were a few isolated cases of HIV/AIDS identified sporadically but the first HIV epidemic in China was not discovered until 1989 when 146 HIV-positive cases were found among intravenous drug users (IDUs) in Ruili, China's border town with Myanmar in Yunnan province [3].

In the early 1990s, the epidemic was mainly along the southwest border regions where more than two-thirds of China's total reported cases of HIV/AIDS originated. The majority of affected people were IDUs, who accounted for about 70% of the country's total reported cases [4]. In the mid-1990s, while the HIV epidemic in border regions slowly spread to nearby regions and inland China, plasma collection activities in central China such as Henan and surrounding provinces caused large numbers of commercial plasma donors to become infected with HIV through infusion of pooled contaminated blood cells [5-7]. The HIV infections among the plasma donor population triggered the second wave of HIV/AIDS epidemic in China. Thereafter a continuous spread of HIV infections among IDU populations persisted. By then, all provinces had reported HIV/AIDS cases, and the epidemic entered a rapid growth phase [4]. The unique characteristics of China's AIDS epidemic in the 1990s were the following: 1) injected drug users and former plasma donors were the two major groups affected by the epidemic, 2) most of the infected patients were young males, and 3) the majority of them lived in rural areas [4].

The large scale blood contamination events among plasma donors were quickly controlled within a short period of time after they were discovered [7]. However, the HIV epidemic among the IDU population remained at fast pace and spread to many parts of the country. There was a steady increase of HIV infections among promiscuous groups in several regions of the country between the late 1990s and early 2000s [8]. The trend of HIV spread in China from high risk groups to the general populations occurred mainly through sexual transmission [8]. According to the joint report by the Ministry of Health, UNAIDS, and WHO, by the end of 2005, there were approximately 650,000 people living with HIV/AIDS in China, among which 75,000 are AIDS patients [9]. New HIV cases are being transmitted primarily through intravenous drug use and risky sexual behavior, indicating that HIV is spreading from the high risk groups to general populations, particular in areas where the epidemic started early [9]. These are all dangerous signs that this terrible disease will expand rapidly unless effective control measures are taken immediately. An early projection predicts 10 millions HIV/AIDS cases in China by 2010 in the absence of effective control measures. The national AIDS control target is set to prevent 85% of potential new HIV/AIDS cases in order to keep the total number below 1.5 millions in China by 2010 [10].

## The social roots of AIDS epidemic in China

The HIV/AIDS epidemic in China has deep roots in a weakened social structure as well as a health infrastructure which are the products of economic reforms from the past two to three decades. Understanding the root causes of these problems is key to finding long-term, effective solutions. For one thing, AIDS must not be treated simply as a medical problem. Social roots for the spread of AIDS include unbalanced development between eastern and western regions of the country; poverty; huge mobile population; inadequate investment in public health; as well as social problems such as drug abuse and prostitution. The most seriously hit population and regions are where social and public health problems are rampant rendering a large number of people vulnerable to infectious diseases. In China, the AIDS epidemic is predominantly among farmers in underdeveloped southwest and northwest border regions as well as inland agricultural provinces. It has been debated in China whether the government should or should not bear the responsibility of providing treatment to AIDS patients. For many years, the AIDS control program in China had only a prevention arm, without treatment and care components. There was no incentive for people to go in for testing, and many people living with HIV/AIDS hid in fear of discrimination.

## Breakthrough in China's AIDS policy

The SARS epidemic in 2003 was a wake-up call for the Chinese government and society as a whole. It has now been widely recognized that public health is not merely a medical issue but rather a security issue, affecting economic growth and social stability. On World AIDS Day 2003, Premier Jiabao Wen announced a new national AIDS control policy, "Four Frees and One Care" (free treatment, free Voluntary Counseling and Testing (VCT), free Prevention of Mother to Child Transmission (PMCT) and free schooling for AIDS orphans, and provision of social relief for HIV patients). By the end of 2005, 20,453 AIDS patients were receiving antiretroviral therapy in 605 counties within 28 provinces. The policy was a breakthrough for China's AIDS control program and had a profound impact even far beyond the AIDS field. The new policy reconnects prevention and treatment, long lost chain in AIDS control work in China. With the government providing free testing and treatment, people living with HIV/AIDS and people at risk are now voluntarily seeking testing and collaborating more frequently with health care workers. Since the adoption of the new policy, there has been a two-fold increase in newly detected cases of HIV/AIDS. In the meantime, more than twelve thousand patients have been treated, and thousands of lives have been saved.

## Current challenges and prospects for future

There are many challenges facing China in implementing the Four Frees and One Care policy and other new initiatives. Bottlenecks impeding rapid and effective implementation of AIDS control programs are no longer political or financial issues. Rather, the greatest problem is the lack of technical capacity, in particular, a shortage of well-trained public health professionals who can manage large scale programs as well as experienced clinical teams who can provide care to patients in rural areas. Even though many early stage HIV/AIDS cases have been identified due to large scale screening programs in the last two years [9], only about 20% of the country's total HIV/AIDS cases are known. Most AIDS patients are in rural areas where medical resources are few and technical capacity is very weak. There are few experienced doctors who are capable of determining toxicity level of HIV/AIDS treatment and adjusting treatment regime accordingly [12]. There is also an urgent need to improve patients' adherence in order to prevent emergence and circulation of drug resistant HIV strains [10,11]. Currently, China has technical capacity to produce only five generic versions of antiretroviral drugs [12]. Consequently, AIDS treatment programs are forced to purchase expensive brand name drugs for first line Anti-Retroviral (ARV) treatment, and thus options for procurement of cost-effective second line drugs need to be explored.

The recent policy breakthrough and new initiatives on AIDS have shown that the Chinese government is fully committed to solving today's public health problems like it did in the 1950s to 1970s. Public health being high on the political agenda has led to significant funding increases and mobilization of public health agencies and local communities. As a result, one can be confident that the AIDS epidemic will be effectively controlled in China in the same way as her past successful history of controlling other infectious diseases. At the same time, what remains concerning is that the national context has changed from a planned economy to a market economy in today's China. Novel disease control measures suitable to the current social and economic structures need to be developed. This can only be achieved through focused research efforts with large groups of scientists specializing in both basic and applied research. China's AIDS research infrastructure is still not adequately established. In ARV treatment and many other AIDS research areas, Chinese researchers have limited experience. Therefore, international collaboration in AIDS research is urgently needed to build up and strengthen China's AIDS research infrastructure. Most of the current international AIDS programs in China do not have a research component. We should encourage both Chinese and international AIDS researchers to work more closely together in the future. Their research collaboration will help China formulate evidence-based treatment, prevention, and control strategies, which can be the bedrock for long-term, effective management of China's national AIDS control programs.
